# Viral Evasion of Innate Immune Defense: The Case of Resistance of Pandemic H1N1 Influenza A Virus to Human Mannose-Binding Proteins

**DOI:** 10.3389/fmicb.2021.774711

**Published:** 2021-12-08

**Authors:** Mitchell R. White, Nikolaos M. Nikolaidis, Francis McCormack, Erika C. Crouch, Kevan L. Hartshorn

**Affiliations:** ^1^Department of Medicine, Section of Hematology and Oncology, School of Medicine, Boston University, Boston, MA, United States; ^2^Division of Pulmonary and Critical Care Medicine, University of Cincinnati, Cincinnati, OH, United States; ^3^Department of Medicine, Washington University School of Medicine, St. Louis, MO, United States; ^4^Department of Pathology & Immunology, Washington University School of Medicine, St. Louis, MO, United States

**Keywords:** influenza, Surfactant protein (SP-D), mannose binding lectin (MBL), cyanovirin, hemagglutin

## Abstract

Mannose-binding lectins effectively inhibit most seasonal strains of influenza A virus and contribute to the innate host defense vs. these viruses. In contrast, pandemic IAV strains are largely resistant to these lectins, likely contributing to increased spread and worse outcomes. In this paper, we evaluated the inhibition of IAV by mannose-binding lectins of human, bacterial, and fungal origin to understand and possibly increase activity vs. the pandemic IAV. A modified version of the human surfactant protein D (SP-D) neck and carbohydrate recognition domain (NCRD) with combinatorial substitutions at the 325 and 343 positions, previously shown to inhibit pandemic H3N2 IAV *in vitro* and *in vivo*, and to inhibit pandemic H1N1 *in vitro*, failed to protect mice from pandemic H1N1 *in vivo* in the current study. We attempted a variety of maneuvers to improve the activity of the mutant NCRDs vs. the 2009 pandemic H1N1, including the formation of full-length SP-D molecules containing the mutant NCRD, cross-linking of NCRDs through the use of antibodies, combining SP-D or NCRDs with alpha-2-macroglobulin, and introducing an additional mutation to the double mutant NCRD. None of these substantially increased the antiviral activity for the pandemic H1N1. We also tested the activity of bacterial and algal mannose-binding lectins, cyanovirin, and griffithsin, against IAV. These had strong activity against seasonal IAV, which was largely retained against pandemic H1N1. We propose mechanisms to account for differences in activity of SP-D constructs against pandemic H3N2 and H1N1, and for differences in activity of cyanovirin vs. SP-D constructs.

## Introduction

Influenza A viruses are a major cause of morbidity and mortality worldwide. The innate immune response plays a key role in the defense against IAVs in the first few days after infection. Pandemic IAV strains pose particular problems since antigenically they are radically different from prior seasonal strains. In addition, pandemic strains, in general, show greater pathogenicity than seasonal strains. Prior studies have shown that innate immunity, in general (Tripathi et al., 2013), and surfactant protein D (SP-D), in particular, play important roles in the early host response to IAVs (Hartshorn et al., 1994; Hawgood et al., 2004). The mechanism of action of SP-D is to bind to high mannose oligosaccharides on the head region of the viral hemagglutinin (HA) in a calcium-dependent manner leading to steric blockade of the HA sialic acid binding site and also to viral aggregation by cross-linking viral particles (Hsieh et al., 2018). Notably, however, pandemic IAV strains (including 1918 and 2009 H1N1s), or recombinant viral strains containing just the HA of a pandemic strain along with the other segments of a seasonal virus, are resistant to SP-D (Job et al., 2010; Qi et al., 2011). Pandemic and avian strains, in general, have limited glycosylation on their HA head regions, which likely accounts for their resistance to inhibition by SP-D. Resistance of pandemic viral strains to inhibition by SP-D or the related collectin, mannose-binding lectin (MBL), is likely one factor in their increased pathogenicity. Avian IAV strains are also generally resistant to inhibition by SP-D due to relative lack of glycosylation on these strains (Parsons et al., 2020). Of note, commonly studied mouse adapted strains like A/Puerto Rico/1934/H1N1 (PR-8) and A/Winston Smith/1931/H1N1 (WSN) fully lack glycosylation on the HA head and are also resistant to collectins.

Pandemic H1N1 of 1918 and 2009 have a single glycan at position 104 on the side of the head region of the HA (see Table 1), which was recently demonstrated to be high in mannose in nature and also critical to sialic acid receptor-binding specificity, which may account for its conservation in natural H1N1 strains (Jayaraman et al., 2012). Seasonal strains of H1N1 during the years after the reintroduction of H1N1 into the human population in 1977 up to 2008 are strongly inhibited by SP-D (Tate et al., 2012). These strains generally have the glycan at 104 but have additional glycans on the HA globular head region. Repeated incubation of the 1979 Brazil H1N1 strain in bovine serum (which contains high concentrations of bovine conglutinin, another collectin) was shown to lead to partial collectin resistance as a result of a loss of the glycan attachment site at 104 by Hartley et al. (1992). Hence, this glycan appears to contribute to attachment of collectins, including SP-D. However, this glycan alone is not sufficient to allow neutralization by SP-D or MBL because the pandemic H1N1 strains are fully resistant.

**TABLE 1:** Major predicted or known glycosylation sites on H1N1 HA molecules of various influenza A virus (IAV) strains and approximate inhibition by wild-type full-length SP-D for each strain.

**Viral strain**	**Type**	**Side of head**	**Top of head**	**Inhibition by SP-D**
NY01 H1N1	Seasonal	104, 286	142, 177	4+
pH1N1 (1918, 2009)*	Pandemic	104	None	0
PR-8 H1N1	Mouse adapted	None	None	0
Braz78 H1N1	Seasonal	104, 286	142, 177	4+
Braz78BS H1N1	Collectin resistant	286	142, 177	3+
IAWSN H1N1	Recombinant, seasonal	104, 286	142, 177	3+

A similar evolution has occurred in human H3N2 viruses, which started out with a high mannose glycan at position 165 on the HA in the original pandemic strains of 1968 but then added additional glycans on the HA head over the years in the human population (Vigerust et al., 2007; Khatri et al., 2016). A bovine serum-resistant variant of the seasonal Philippine 1982 H3N2 strain lacks both the glycan at 165 and another high mannose glycan on the head of the HA at position 246 (Khatri et al., 2016). It has been shown that HA glycosylation is protective vs. neutralizing antibody binding while also making the seasonal viruses more susceptible to host defense lectins like SP-D or MBL (Job et al., 2013). In contrast to the pandemic H1N1 strains, however, the 1968 H3N2 viruses are partially inhibited by these lectins suggesting that the high mannose glycan at position 165 is either more accessible or better positioned to allow some inhibition by SP-D and other lectins or that other high mannose oligosaccharides may be present on the HA (Nikolaidis et al., 2014).

Modification of innate defense mediators such as collectins and antimicrobial peptides can result in more effective inhibition of IAVs. We and others have studied the activity of recombinant versions of SP-D composed of only the neck and carbohydrate recognition domains (NCRDs) of the molecule, without the extended collagen domain or N-terminus (Clark et al., 2003; Knudsen et al., 2007; Crouch et al., 2009, 2011; Hartshorn et al., 2010; White et al., 2010). The ability to crystallize NCRDs and perform structure–function analysis make them useful for determining mechanisms of viral binding and neutralization. The NCRD of wild-type human SP-D (wild-type NCRD) has glycan-binding activity and protective effects in mouse models of allergy and respiratory syncytial virus infection, but has minimal activity vs. IAVs (Nikolaidis et al., 2014). This appears to result from loss of cooperative binding effects afforded by having multiple trimeric NCRD heads on the full-length SP-D molecule (Tecle et al., 2008; White et al., 2010). We have reported, however, that mutating residues surrounding the lectin site of the SP-D NCRD confers increased viral binding and neutralizing activity. In particular, a mutant form of the trimeric carbohydrate recognition domain (NCRD) of SP-D with combinatorial substitutions at the 325 and 343 positions (D325A or S or R343V) has greatly increased activity against seasonal IAV *in vitro* and *in vivo* (Goh et al., 2013). The double mutant improved survival of mice infected with pandemic H3N2 (Aichi68) compared with the wild-type NCRD. In addition, double mutant NCRDs had the ability to inhibit pandemic strains of H1N1 *in vitro* (Nikolaidis et al., 2014). In this paper, we demonstrated, however, that the modified versions of NCRD were not able to protect mice against infection with pandemic H1N1 of 2009, and we explored avenues of increasing antiviral activity and compared the activity to other mannose-binding lectins of human, bacterial, or algal origin.

## Materials and Methods

### Virus Preparations

The A/Philippines/1982/H3N2 (Phil82) and A/Brazil/1978/H1N1 (Braz78) strain, and their bovine serum inhibitor-resistant variants (Phil82BS and Braz78BS), as well as the A/Memphis/1971/H3N1 (Memphis71) strain were kindly provided by Dr. E. Margot Anders (University of Melbourne, Melbourne, Australia). The A/Aichi/1968/H3N2 (Aichi68) strain, A/Wyoming/2003/H3N2 (Wyoming03), and the murine parainfluenza virus Sendai/52 were obtained from the American Tissue Type Collection (ATCC) (Manassas, VA, United States). The PR-8 strain was kindly provided by Dr. Jon Abramson (Wake Forest University). These IAV strains were grown in the chorioallantoic fluid of 10-day-old chicken eggs and purified on a discontinuous sucrose gradient as previously described (Hartshorn et al., 1988). The virus was dialyzed against PBS to remove sucrose, aliquoted, and stored at −80°C until needed. Post-thawing, the viral stocks contained ∼5 × 10^8^ infectious focus forming units/ml. For HA inhibition experiments, several additional egg-grown strains were used including the A/California/2009/H1N1 pandemic strain (Cal09) and the A/New York/2001/H1N1 (NY01) seasonal strain, which were prepared by reverse genetics as described (Qi et al., 2011). The A/WSN/1933/HAnc-AspGly/H1N1 strain (Ia WSN) was also produced by reverse genetics and included the HA of a seasonal H1N1 strain (with a modification to allow binding to alpha 2–3-linked sialic acids to allow replication in mice) combined with the other proteins of the WSN virus (Smee et al., 2008). This strain was graciously provided by Dr. Donald Smee (Utah State University). The reverse genetics-derived strains were grown in MDCK cells as described. Table 1 shows the positions of glycan attachment sites on the head region of the HA of H1N1 viral strains used in this paper.

### Collectin and Bacterial or Algal Lectin Preparations

Recombinant human SP-D trimers, dodecamers, and multimers were produced in CHO cells as previously described (Hartshorn et al., 1996). Full-length SP-D trimers, dodecamers, and multimers containing the D325A + R343V mutations in the CRD were prepared in the same manner. Trimeric NCRD fusion proteins consisting of the neck domain and carbohydrate recognition domains of SP-D were combined with N-terminal tags that contain a His-tag and S-protein-binding site that permit purification and/or detection. R343V and D325A + R343V were produced and characterized as previously described (Crouch et al., 2011). The D325A + F335Y + R343V fusion protein was produced in the same way. All showed a single major band of appropriate size by SDS-PAGE and demonstrated the expected decrease in mobility on reduction, consistent with appropriate formation of intrachain disulfide bonds. The endotoxin level of all SP-D preparations was 0.1 ∼ 0.5 EU/ml (Limulus Lysate Assay, Cambrex, Walkersville, MD, United States).

Cyanovirin and scytovirin are derived from cyanobacteria, and griffithsin is derived from red algae and all are mannose-binding lectins with the ability to inhibit HIV and a variety of other viral pathogens (Hu et al., 2007; Jensen et al., 2014). These were prepared by Smee et al. (2008).

### Hemagglutination Inhibition Assay

Hemagglutination (HA) inhibition was measured by serially diluting collectins in round bottom 96-well plates (Serocluster U-Vinyl plates; Costar, Cambridge, MA, United States) using PBS containing calcium and magnesium as a diluent and human type O red cells as described (Hartshorn et al., 1994).

### Measurement of Viral Aggregation

Viral aggregation caused by collectins was measured by assessing light transmission through stirred suspensions of IAV as described (Hartshorn et al., 1994). This was done using a Perkin Elmer Lambda 35 UV/Vis spectrophotometer. In addition, viral aggregation was assessed using electron microscopy (EM) as described (Tecle et al., 2008).

### Fluorescent Focus Assay and Plaque Assay of Influenza A Virus Infectivity

MDCK cell monolayers were prepared in 96-well plates and grown to confluency. These layers were then infected with diluted IAV preparations for 45 min at 37°C in PBS (with 2 mM calcium and magnesium). MDCK cells were tested for the presence of IAV-infected cells after 7 h of virus addition using a monoclonal antibody directed against the influenza A viral nucleoprotein (provided by Dr. Nancy Cox, CDC, Atlanta, GA, United States). IAV was preincubated for 30 min at 37°C with various concentrations of collectins or control buffer, followed by the addition of these viral samples to the MDCK cells. Infected cells (i.e., fluorescent cells) in each well of the 96-well plates were counted after 7 h, and infectious particles/ml in the suspension were determined from that. This assay has been shown to correlate well with viral plaque assays (Reading et al., 1997).

### Murine Model of Influenza A Virus Infection

**FIGURE 1:**
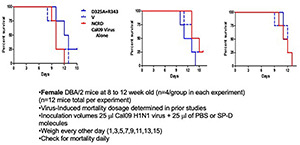
Mouse survival after infection with Cal09 H1N1 influenza A virus (IAV). The viral inoculum was instilled along with the D325A + R343V mutant surfactant protein D (SP-D) neck and carbohydrate recognition domain (NCRD) (D325A + R343V), wild-type SP-D NCRD (NCRD), or an equal quantity of PBS (Cal09 virus alone). There were no differences in mortality among the groups. The methodology for these experiments is outlined below the figures.

**FIGURE 2:**
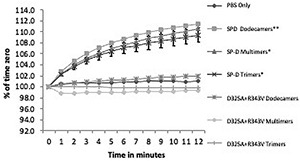
Aggregation of Phil82 H3N2 virus by full-length wild-type or D325A + R343V molecules. Aggregation of the virus was assessed by light transmission, and 2 μg/ml of SP-D molecules was added at time 0. Wild-type SP-D multimers, dodecamers, and trimers all caused significant viral aggregation (^∗^*p* < 0.05 vs. control and ^∗∗^*p* < 0.01 vs. control) (mean ± SEM; *n* = 5). The full-length multimers, dodecamers, and trimers containing the D325A + R343V mutations did not cause significant aggregation.

An outline of the murine experiments is given in Figure 1. *In vivo* mortality studies were conducted with 10- to 16-week-old female DBA/2J mice (The Jackson Laboratory, Bar Harbor, ME, United States) as previously described (Crouch et al., 2011). These mice were chosen due to their increased susceptibility to IAV infection. Mice were lightly anesthetized with isoflurane. Fifty microliters of *Cal09* stock in PBS with Ca^2+^ and Mg^2+^ ions was co-administered *via* oropharyngeal aspiration with or without 20 μg/mouse of D325A + R343V NCRD or wild-type NCRD. The Cal09 virus used was grown in MDCK cells and used directly as a culture supernatant from those cells. Initial experiments were performed to determine the LD50 of this preparation for the mice (data not shown). Viral titers of the inoculum used for the studies shown in Figure 2 were with 1.5 × 10^3^ viral particles/ml, as assessed by real-time quantitation (AD502, iGentBio San Diego, CA, United States). Similar results were obtained by infectious focus assays. Mice were weighed every other day and checked daily for vital status. All animals were maintained in a specific pathogen-free facility and were handled according to an institutional animal care and use committee (IACUC)-approved protocol and the National Institutes of Health guidelines.

### Statistics

Statistical comparisons were made using Student’s paired, two-tailed *t*-test or ANOVA with *post hoc* test (Tukey’s). ANOVA was used for multiple comparisons to a single control.

## Results

### *In vivo* Activity of Neck and Carbohydrate Recognition Domains vs. Cal09

Cal09 H1N1 is a highly lethal viral strain in murine studies, which contrasts with seasonal human viruses that must undergo mouse adaptation or be inoculated at very high concentrations to cause lethality. As shown in Figure 1, D325A + R343V did not provide protection for mice against the Cal09 H1N1 strain either in terms of mortality. The results shown are from three independent experiments. There was also no difference in weight loss among the different treatment groups in these experiments (data not shown). These results contrast with our prior findings with a strain containing a seasonal H1N1 HA (Ia WSN; see Section “Materials and Methods” for description) and the pandemic H3N2 Aichi strain (Crouch et al., 2011; Nikolaidis et al., 2014).

### *In vitro* Activity of Full-Length Surfactant Protein D Proteins Containing D325A + R343V Mutations in the Carbohydrate Recognition Domain

**FIGURE 3:**
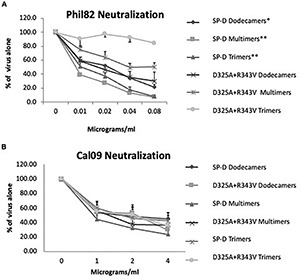
Viral neutralization by full-length wild-type or D325A + R343V molecules. These experiments were performed using the same preparations as in Figure 2 in viral neutralization assays. Neutralization in this and subsequent assays were tested by pre-incubating virus inoculums with the various inhibitory proteins followed by measuring virus titers with the infectious focus assay *in vitro* (see “Materials and Methods”). Neutralization activity against Phil82 H3N2 is shown in **(A)**. Instances of significant inhibition compared with control are indicated by ^∗^ for *p* < 0.05 and ^∗∗^ for *p* < 0.01 (mean ± SEM; *n* = 4). Neutralization activity against Cal09 is shown in **(B)**. Note the full-length proteins containing the D325A + R343V mutations caused greater neutralization than wild-type SP-D molecules.

A full-length protein containing the D325A + R343V mutations was developed hoping to see increased activity compared with NCRD. The full-length protein contains the N-terminal and collagen domains of the molecule missing in the NCRDs and is capable of forming multimers of various sizes (trimers, dodecamers, and high molecular weight multimers). Full-length SP-D has much greater antiviral activity against seasonal strains than wild-type SP-D NCRD, and this activity increases with increased levels of multimerization of the full-length protein. We tested trimeric, dodecameric, and multimeric forms of the mutant protein against seasonal and pandemic strains of IAV. Our expectation was that the full-length mutant proteins would cause marked aggregation of IAV since they could combine the aggregating activity of the mutant NCRD with that of the multimeric full-length protein. However, as shown in Figure 2, the full-length mutant proteins had substantially diminished aggregating activity compared with the wild-type SP-D (Phil82 seasonal strains were used in these experiments). As shown in Figure 3A, the full-length mutant protein did not have increased activity against the seasonal Phil82 IAV on neutralization assays when compared with equally multimerized forms of wild-type human SP-D. In fact, the trimer and multimer forms had significantly less neutralizing activity than wild-type SP-D. Nearly 100-fold higher doses of the proteins were used in neutralization assays with Cal09, since lower doses of full-length SP-D as used in the Phil82 experiments show no activity (Figure 3B). In these experiments, all of the proteins had roughly similar neutralizing activity against Cal09.

### Effect of Cross Linking of Neck and Carbohydrate Recognition Domains on Antiviral Activities Against Cal09 Versus Other Viruses

**FIGURE 4:**
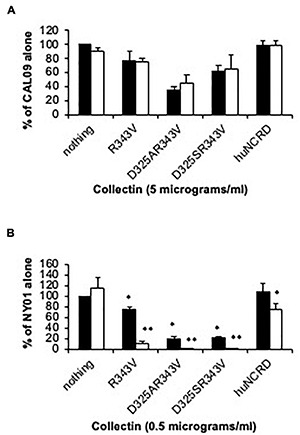
Effect of cross-linking NCRD trimers on neutralizing activity against pandemic vs. seasonal H1N1 viruses. The ability of the indicated NCRD trimers to neutralize either Cal09 H1N1 (labeled rA/CA; pandemic) or NY01 (labeled rNY312; seasonal strain from 2001) was tested **(A,B)** respectively. The NCRDs were used alone (black bars) or after cross-linking with anti-SP-D antibody (white bars), whereas the antibody potentiated activity of the NCRDs vs. seasonal NY01 (^∗∗^*p* < 0.05 vs. NCRD alone) did not have this effect vs. Cal09 (*n* = 4 experiments for NY01 and *n* = 5 for Cal09, mean ± SEM). (^∗^*p* < 0.05 vs. virus alone).

We have previously demonstrated that cross-linking of wild-type NCRD or R343V within SP-D NCRD-specific mAb significantly increases neutralizing activity against seasonal strains of IAV (Tecle et al., 2008). These maneuvers did not, however, increase activity against Cal09 (Figure 4A). As an internal control, we used a seasonal H1N1 viral strain (NY01), which was prepared by reverse genetics and grown in MDCK cells like Cal09. Both cross-linking maneuvers increased the activity of the NCRDs against NY01 (Figure 4B).

### Effect of Combining Alpha 2 Macroglobulin With Surfactant Protein D or Neck and Carbohydrate Recognition Domains on Neutralization of Cal09 H1N1

**FIGURE 5:**
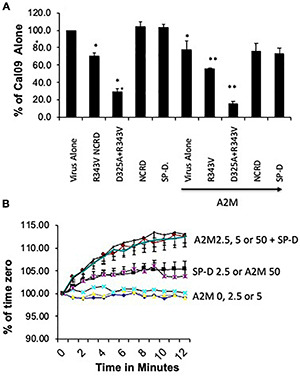
Effect of alpha 2 macroglobulin (A2M) alone or in combination with SP-D proteins on neutralizing activity vs. Cal09 H1N1 or aggregation of Phil82 H3N2. **(A)** The NCRDs (at 1 μg/ml) or A2M (at 5 μg/ml) were added alone or in combination with the Cal09 strain, and neutralizing activity was measured (^∗^*p* < 0.05 vs. control and ^∗∗^*p* < 0.05 vs. control or collectin proteins or A2M alone; mean ± SEM; *n* = 4). **(B)** The ability of full-length SP-D dodecamers alone (2.5 μg/ml) and A2M (2.5, 5, or 50 μg/ml) were added alone or in combination to stirred suspensions of the Phil82 strain to test for viral aggregation. A2M alone at 2.5 or 5 μg/ml did not cause any viral aggregation, although increasing A2M concentration to 50 μg/ml did. SP-D caused viral aggregation. Combining SP-D with A2M caused increased aggregation compared with SP-D alone (all combinations *p* < 0.05 vs. either SP-D or A2M alone; mean ± SEM; *n* = 4).

A2M has been reported to bind to SP-D, and this interaction increases the ability of SP-D to aggregate bacteria (Craig-Barnes et al., 2010). Furthermore, A2M has been reported to be an essential element in saliva for inhibiting pandemic H1N1 (Chen et al., 2010). We therefore combined A2M with SP-D dodecamers or NCRDs in hopes of increasing neutralization of Cal09. A2M did have a slight neutralizing activity for Cal09 and had a slight additive activity with the mutant NCRDs (Figure 5A). In the figure, ^∗^ indicates where A2M or collectin alone reduced viral infectivity compared with the control, and ^∗∗^ indicates where combinations of A2M with collectins caused significantly greater reduction of infectivity compared with A2M or the respective collectin alone (*p* < 0.05). Adding A2M also strongly increased the ability of SP-D to aggregate Phil82 IAV, even at concentrations of A2M that did not induce viral aggregation on their own (Figure 5B). These results suggest that binding of A2M to SP-D does not inhibit its interaction with the IAV.

### Effect of Introducing an Additional Mutation in the Neck and Carbohydrate Recognition Domains on Antiviral Activity

**FIGURE 6:**
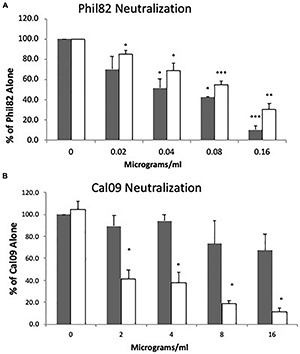
Neutralizing activity of double mutant D325A + R343V vs. triple mutant D325A + F335A + R343V. We tested the neutralizing activity of the noted NCRDs against Phil82 **(A)** or Cal09 **(B)**. D325A + R343V is shown by black bars, and D325A + F335A + R343V is shown by white bars. Both NCRDs caused significant inhibition of the Phil82 strain in the ng/ml range (^∗^*p* < 0.05, ^∗∗^*p* < 0.01, and ^∗∗∗^*p* < 0.005). In these experiments the triple mutant D325A + F335A + R343V caused significant inhibition of the Cal09 strain in μg/ml range (^∗^*p* < 0.05 vs. control; mean ± SEM; *n* = 5).

We previously showed that the single site mutant NCRD, F335Y, had some increase in mannan-binding activity against Phil82 compared with the wild-type NCRD. In addition, F335 was shown to participate in binding of SP-D to extended carbohydrate chains, which was retained in the F335Y mutant (Crouch et al., 2006). We therefore constructed a triple mutant NCRD, D325A + F335Y + R343V. This construct had strong viral neutralizing activity as well; however, the activity was significantly less than that of D325A + R343V for the Phil82 IAV (Figure 6A). Of interest, the triple mutant construct did have increased activity against Cal09 *in vitro* compared with D325A + R343V (Figure 6B).

### Activity of Bacterial Mannose-Binding Lectins vs. Seasonal and Pandemic Influenza A Virus Strains

Cyanovirin has specifically been shown to inhibit IAV through binding to high mannose lectins on the HA (Smee et al., 2007). We did screening tests on the activity of these lectins against a panel of IAV strains using hemagglutination inhibition assays (Table 2). Cyanovirin and griffithsin had potent anti-IAV activity against seasonal strains of IAV but lacked activity against the mouse-adapted strain PR-8 (which lacks high mannose glycans on its HA head) or Phil82BS. Importantly, the loss of activity of these lectins against Phil82BS was profound, indicating that the high mannose glycans at 246 and 165 (which are missing in Phil82BS) are critical for their binding to the HA. Scytovirin had minimal activity against the Phil82 H3N2 strain but not against the other strains tested. Cyanovirin was consistently more active than griffithsin in these assays. Of interest, the activity of cyanovirin was highest against Braz79 with partial loss of activity against Braz79BS indicating a role for the glycan at position 104 (missing in Braz79BS) on the H1 HA in binding to cyanovirin. Results with SP-D and MBL from concurrent assays are shown for comparison.

**TABLE 2:** Hemagglutinin (HA) inhibition of IAV strains by bacterial and human lectins.

	**Cyanovirin**	**Griffithsin**	**Scytovirin**	**Surfactant protein D (SP-D)**	**Mannose-binding lectin (MBL)**
Phil82 H3N2	0.11 ± 0.002	0.27 ± 0.08	23.5 ± 7.7	0.017 ± 0.005	0.058 ± 0.001
Phil82/BS H3N2	>25	>25	>25	0.157 ± 0.01	0.46 ± 0.04
Braz79 H1N1	0.044 ± 0.012	0.39 ± 0.2	>2.5	0.019 ± 0.004	0.019 ± 0.004
Braz79/BS H1N1	0.1 ± 0.027	0.85 ± 0.56	>2.5	0.022 ± 0.004	0.029 ± 0.009
Wyoming03 H3N2	0.04 ± 0	0.17 ± 0.07	>2.5	0.038 ± 0.01	0.029 ± 0.009
Memphis71 H3N1	0.36 ± 0.09	>2.5	>2.5	0.22 ± 0	
PR-8 H1N1	>25	>25	>25	>25	>25
Sendai52	>2.5	>2.5	>2.5	0.67 ± 0.17	>2.5
IAWSN H1N1	1.5 ± 0.06	>25	>25	0.24 ± 0.01	

*HA inhibition was tested using human type O red cells as described. Results are mean ± SEM of a minimum of three experiments in each case.*

*Full names of the viral strains are provided in the Section “Materials and Methods.” Note that Sendai52 is a murine parainfluenza virus.*

*Results show minimum concentration of inhibitor needed to block one HA unit of IAV in μ/ml.*

**FIGURE 7:**
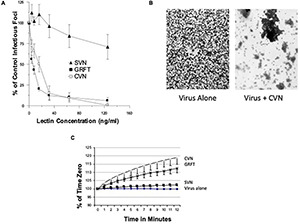
Evaluation of neutralizing activity and viral aggregating activity of cyanovirin, griffithsin, or scytovirin. **(A)** Neutralizing activity of the indicated collectins (cyanovirin = CVN, griffithsin = GRFT, and scytovirin = SVN) was tested vs. the Phil82 strain of IAV (mean ± SEM; *n* = 5). **(B)** The ability of cyanovirin to aggregate this viral strain was assessed by electron microscopy (representative experiments of three). Aggregation by the three lectins was tested also by light transmission in **(C)** (mean ± SEM; *n* = 4).

**FIGURE 8:**
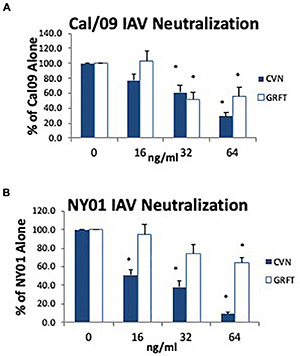
Neutralizing activity of cyanovirin and griffithsin against Cal09 H1N1 or NY01 H1N1 viruses. The ability of cyanovirin and griffithsin to neutralize Cal09 **(A)** or NY01 **(B)** was tested at the indicated concentrations in ng/ml (^∗^*p* < 0.05 vs. control; mean ± SEM; *n* = 5).

Cyanovirin, griffithsin, and scytovirin were also tested in neutralization and aggregation assays against Phil82 (Figure 7A). Cyanovirin and griffithsin induced viral aggregation of Phil82 (Figures 7B,C) (confirmed for cyanovirin by electron microscopy, Figure 7B), suggesting the importance of multivalent binding in antiviral activity. As shown in Figure 8, both cyanovirin and griffithsin had neutralizing activity against Cal09, which was unexpected. In fact, their activity against Cal09 was comparable with their activity against Phil82.

## Discussion

Our prior studies revealed that R343V and D325A + R343V have a different mode of binding to extended mannose chains in that of the wild-type NCRD. The mutant proteins bind to the penultimate mannose in the chain, while wild-type NCRD binds to the terminal mannose (Goh et al., 2013). As a result of this, the mutant proteins can more fully cover up the sialic acid binding site of the HA and to bind to the HA in such a way that the other two lectin sites on the CRD trimer remains exposed so they can more easily attach to other HA molecules. The latter observation provides a potential explanation for viral aggregation as mediated by the mutant NCRDs (in contrast to wild-type NCRD). Both blocking of the sialic acid binding site and ability to induce viral aggregation appear to be important for lectin-mediated inhibition of IAV by collectins, and this appears to be the case for cyanovirin and griffithsin as well, based on our current findings.

As noted, our studies of the Phil82 and Phil82BS strains indicated the importance of two high mannose glycans on the H3 HA head for neutralization by SP-D (Khatri et al., 2016). These studies also showed that the Phil82/BS strain lacks two high mannose glycans on the exposed tip of the HA (those at position 165 and 246), and implicated that both of these sites are needed for optimal binding by SP-D. This latter conclusion was based on molecular dynamics evaluations showing that these two glycans are positioned such that the three lectin sites on the SP-D trimer can attach simultaneously to various combinations of the two glycans. Note that the pandemic H3N2 strain of 1968 had the glycan at 165 but lacked several other potential glycan attachment sites on its HA. This may account for the reduced ability of wild-type SP-D to inhibit this strain compared with the more recent H3N2 strains including Phil82.

Phil82 and other seasonal human IAV strains do not replicate well in mice or cause mortality. This likely reflects its high level of sensitivity to endogenous SP-D and the specificity of its HA for human sialic acid-binding receptors on airway cells (which differ in mice). In contrast, pandemic H3N2 of 1968 (Aichi) does cause mouse mortality, which could be overcome by D325A + R343V but not by wild-type NCRD (Nikolaidis et al., 2014). Our initial expectation was that D3254A + R343V would show protective activity in mice against Cal09. As noted, the 1918 and 2009 H1N1 pandemic strains both have one glycan attachment site at position 104 (shown to be of high mannose for 2009 H1N1). However, we now show that D325A + R343V NCRD did not protect mice against Cal09 H1N1. We cannot exclude the possibility that lesser levels of protection might be seen *in vivo* [e.g., reduced cytokine production or viral loads as reported for SP-D *in vitro* (Al-Ahdal et al., 2018), but at least, by the stringent criteria of mortality and weight loss used in our prior studies, we did not see protection].

One simple explanation for the failure of D325A + R343V to protect mice against Cal09 strain is the relative difference in its neutralizing activity for Aichi68 and Cal09. To achieve ≈80% inhibition of infectivity, there is an approximately 10-fold difference for the two strains (i.e., ≈1 μg/ml for Aichi68 and ≈10 μg/ml for Cal09). We decided to explore additional measures to increase lectin activity against Cal09 *in vitro*. Unfortunately, creation of full-length DR molecules did not result in increased activity. This was surprising given the markedly greater antiviral activity of full-length multimerized SP-D compared with the wild-type SP-D NCRD. One possible interpretation of this is that the advantages of the D325A + R343V NCRD are diminished in the full-length molecule. For instance, the ability to assume a different orientation in relation to the viral HA (compared with wild-type NCRD) may not be possible in the full-length molecule leading to impaired ability to induce viral aggregation.

Cross-linking of wild-type and mutant NCRDs with some anti-SP-D antibodies increases activity against seasonal strains of IAV. Unfortunately, this was again not the case for the Cal09 strain. Since α2 macroglobulin (A2M) was shown to bind to SP-D and increase antibacterial activity (Craig-Barnes et al., 2010), we tested if this combination would increase activity of Cal09. There was slight antiviral activity for A2M, and additive effects when combining it with SP-D in antiviral assays vs. Cal09. The combination of SP-D and A2M also resulted in a significant increase in viral aggregation compared with SP-D alone as well. These results confirm an interaction of SP-D and A2M. However, the level of activity against Cal09 would not be expected to have a substantial impact *in vivo*.

Another approach is to further engineer the NCRD of SP-D to increase lectin activity. We did try an introduction of a third mutation, F335Y, to the D325A + R343V mutant based on modeling studies and mannose-binding studies of the F335Y mutant. The introduction of a third mutation to D325A + R343V did not increase activity against seasonal IAV (Phil82), but the triple mutant did have significant activity against Cal09, although this was still in the μg/ml range and not the ng/ml range of activity needed for strong *in vivo* inhibition. We do not feel that this excludes the possibility that other changes in the NCRD will increase activity for pandemic strains. Studies of porcine SP-D are instructive in this regard. Porcine SP-D differs from human or rodent SP-D in two major regards. It has an N-linked sialylated glycan on its CRD, which does not exist in other SP-Ds and provides an additional method of attaching to HA (i.e., binding of the HA to sialic acids on this glycan) (van Eijk et al., 2004, 2011), and it has several key amino acid changes in the NCRD that independently confer an increase in viral neutralizing activity compared with human or rodent SP-Ds (van Eijk et al., 2012). Recently, van Eijk et al. (2019) showed that introducing these key amino acid changes in porcine SP-D into full-length human SP-D resulted in a protein with increased potency and broadened spectrum of activity against pandemic and avian IAV strains, even without the N-linked glycan present on porcine SP-D. Those findings indicate that SP-D molecules modeled after porcine SP-D are a promising route to increasing activity against pandemic IAV.

We thought that the study of cyanovirin, griffithsin, and scytovirin might be revealing with regard to a potential of different mannose-binding lectins to inhibit pandemic strains. Cyanovirin showed a level of HA inhibitory activity against a panel of IAV strains comparable with SP-D. This agrees with prior findings *in vitro* and *in vivo* in mice with cyanovirin (Smee et al., 2008). Griffithsin has been shown to inhibit a number of enveloped viruses, but to our knowledge, this is the first demonstration of antiviral activity for IAV (Alexandre et al., 2020). The activity of griffithsin was somewhat less than that of cyanovirin, in general. We found only minimal activity for scytovirin and only for the Phil82 strain, and not the various H1N1 strains or other H3N2 strains. These lectins have been shown to bind high mannose structures, and for cyanovirin, loss of N-linked glycosylation sites 104 on seasonal H1N1 (1999 New Caledonia strain) was shown to confer resistance. We found only a partial loss of activity of cyanovirin or griffithson in the Braz79BS strain. There was, however, a very marked loss of activity for these proteins for the Phil82BS strain. This exceeded the loss of activity of SP-D or MBL for this strain. Of most interest, however, cyanovirin and griffithsin had significant antiviral activity against Cal09. This activity was somewhat less than its activity against a seasonal H1N1 strain (NY) but greater than the activity of the optimal collectin mutant D325A + R343V.

The finding that cyanovirin and griffithsin are able to inhibit Cal09 more effectively suggests that their mechanism of attachment or viral crosslinking differ significantly from the collectins. It is worthwhile to note in this context that these lectins have strong activity against HIV, whereas collectins do not (Meschi et al., 2005). For instance, we found that a concentration of 5 μg/ml of SP-D was needed to approach 50% inhibition of infectivity of HIV *in vitro* (compared with ng/ml concentrations of SP-D to inhibit IAV or ng/ml concentrations of cyanovirin or griffithsin to inhibit HIV) (Lee, 2019). These findings also suggest differences in the mechanisms of action of collectins vs. cyanovirin and griffithsin. This could be elucidated by structural binding studies. In any case, it is possible that cyanovirin or related compounds may be another promising avenue for treatment or prevention of pandemic IAV.

It appears that with the important exception of porcine SP-D or the porcinized human SP-D noted above, the ability of SP-D (or MBL) to inhibit IAV depends on the presence of high mannose oligosaccharides on or near the globular domain of the HA. The reasons why some glycans remain predominantly high in mannose on the HA are not fully clear, but one factor is lack of accessibility of those glycans to the glycan-processing machinery in the ER and Golgi apparatus. A recent nice demonstration of this was the finding by Parsons et al. (2020): a glycan in a similar position to the high mannose glycan at 165 is present in several avian Has, including H2, H5, H6, and H11 avian Has. However, these glycans are of complex type and do not allow for binding or inhibition by SP-D. Human and an avian H3-containing virus did allow binding of SP-D. Importantly, it was shown that differences in amino acid sequence and structure of the H2 and H6 has resulted in far fewer contacts between the N165 glycan and the protein surface than is seen with the H3 HA and further that. Comparison of amino acid structures of the HA can allow prediction of which ones will have high mannose glycans and, hence, be susceptible to inhibition by SP-D. This also appears to be one factor determining the severity of infection by novel viral strains, although other properties of the HA of different strains and subtypes determine pathogenicity (Qi et al., 2014), and other viral genes play a role in pathogenicity as well (e.g., the NA and PB1 genes of the 1918 virus) (Kobasa et al., 2007).

**FIGURE 9:**
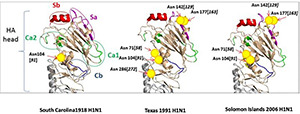
Ribbon diagram of H1N1 pandemic and seasonal strains showing location of glycosylation sites. This figure was obtained with permission from Sun et al. (2013), and it shows comparative structures of the head regions of the hemagglutinin (HA) of H1N1 strains from 1918 (pandemic), 1991 (seasonal), and 2006 (seasonal). Glycosylation sites are indicated by yellow circles. Sa, Sb, Ca, and Cb represent antigenic areas of the HA. Glycosylation sites on the top of the head of the HA (e.g., 142 or 177), which are found on seasonal H1N1 strains and missing on pandemic strains (including 1918 and 2009), can interfere with antibody recognition of antigenic sites but also provide binding sites for SP-D. The numbers indicated by parenthesis represent alternative numbering sometimes used for glycosylation sites.

The positioning of high mannose glycans may make them more or less accessible to attachment by mannose-binding lectins, and the glycan at position 104 on the H1N1 HA is likely less accessible than the glycan at 165 position on H3N2 since the 165 glycan is positioned higher up on the HA head compared with the 104 glycan, which is lower down on the side of the HA head (Figure 9). For Braz79 on other recent seasonal H1N1 strains like NY01, other high mannose glycans on the HA head contribute importantly to binding by SP-D (noting relatively minor loss of HA-inhibiting activity for Braz79/BS for mannose-binding lectins). Sun et al. (2013) have shown that adding glycans at 144 and 172 to the 1918 HA head (as seen in seasonal H1N1 strains) like Braz79 leads to decreased pathogenicity in mice. Note that these glycans are positioned on the distal tip of the HA (like 165 in H3 HA) (see Figure 9). We conclude that the collectins need more than one high mannose glycan on the H1 HA head region for optimal neutralization, and optimally, these glycans are on the distal tip of the HA. A similar case occurs with the H3 viruses. Further studies of Braz79 HA glycan structure are underway and may help explain the failure of collectins to inhibit the pandemic H1N1 strains sufficiently to provide protection.

Both the H3 and H1 subtypes of IAV have established long survival in the human population through their abilities to evade immune recognition through antigenic drift over time. Both of these subtypes have added glycans (including more high-mannose glycans) on the HA over time compared with their original pandemic strains. This seems paradoxical since these strains become also more susceptible to inhibition by mannose-binding lectins. A very interesting recent paper (Altman et al., 2019) found that initial H1, H2, and H3 pandemic strains have few glycan attachments but that glycans were added to the HA head region of H1 and H3 viruses at ∼5- to 6-year intervals to a maximum number of approximately six to seven glycans. After this, glycan positions are swapped at a slower rate. Addition of new glycans correlates with immune escape and dominance of the new strain, which is not recognized by prior HA-specific antibodies. Addition of glycans is limited by their tendency to reduce receptor binding affinity. In contrast, the H2 HA [which is not inhibited by SP-D (Hartshorn et al., 2008)] was more limited in its ability to add glycans without major loss of binding and fusion activity of the HA and had a shorter period of circulation in humans than H1 or H3 viruses. Hence, IAV strains in the human population must balance the ability to evade antibody-mediated immunity by masking epitopes (or other means) and either losing receptor binding affinity or becoming susceptible to inhibition by host defense lectins or other innate inhibitors.

Overall, our studies suggest that there are limits to the ability of human SP-D, even with some of the modifications we have introduced, to inhibit some pandemic and most avian strains of IAV. As noted, however, recent studies of porcinized human SP-D are more encouraging since this seems to have wide ranging activity against various IAV strains, including pandemic and avian strains. In addition, presence of a sialylated glycan on porcine SP-D could be exploited to broaden antiviral activity. Our findings with cyanovirin and griffithsin are encouraging as well since these seem to retain substantial activity against pandemic H1N1. SP-D has been shown to inhibit SAR-Cov1 in prior studies (Leth-Larsen et al., 2007), and a recent paper showed that a recombinant NCRD fragment of SP-D also inhibits SAR-Cov2, which has a heavily glycosylated spike protein (Vankadari and Wilce, 2020; Hsieh et al., 2021). Hence, evaluating SP-D and the modified collectins or other mannose-binding lectins for activity against the novel virus may be worthwhile.

## Data Availability Statement

The original contributions presented in the study are included in the article/supplementary material, further inquiries can be directed to the corresponding author.

## Ethics Statement

The studies involving human participants were reviewed and approved by IRB, Boston Medical Center. The patients/participants provided their written informed consent to participate in this study. The animal study was reviewed and approved by IACUC, Cincinnati University School of Medicine.

## Author Contributions

MW contributed to experimental plan, performance of experiments, and preparation of manuscript. NN and FM contributed to experimental plan and performance of experiments. EC contributed to experimental plan, performance of experiments, and development and production of SP-D proteins. KH contributed to experimental plan, performance of experiments, and writing of manuscript. All authors contributed to the article and approved the submitted version.

## Conflict of Interest

The authors declare that the research was conducted in the absence of any commercial or financial relationships that could be construed as a potential conflict of interest.

## Publisher’s Note

All claims expressed in this article are solely those of the authors and do not necessarily represent those of their affiliated organizations, or those of the publisher, the editors and the reviewers. Any product that may be evaluated in this article, or claim that may be made by its manufacturer, is not guaranteed or endorsed by the publisher.
